# Supplementary material for: Identification of photoperiod-induced specific miRNAs in the adrenal glands of Sunite sheep (Ovis aries)
Source: Front Vet Sci. 2022 Jul 22;9:888207. doi: 10.3389/fvets.2022.888207 (PMC9354845; doi:10.3389/fvets.2022.888207)
Supplement: Supplementary file 1 [file Data_Sheet_1.ZIP › Supplementary file/miRDeep2 file.zip!/miRDeep2 file/Novel_Structure/LP42A1/Novel_206.pdf]

Provisional ID	:	NC_019466.2_17133
Score total	:	2.5
Score for star read(s)	:	-1.3
Score for read counts	:	0
Score for mfe	:	2.2
Score for randfold	:	1.6
Score for cons. seed	:	
Total read count	:	14
Mature read count	:	14
Loop read count	:	0
Star read count	:	0

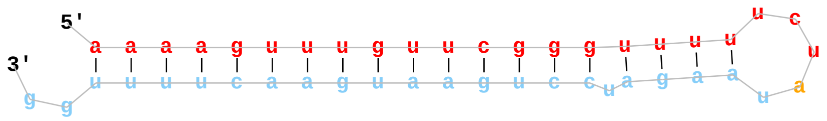

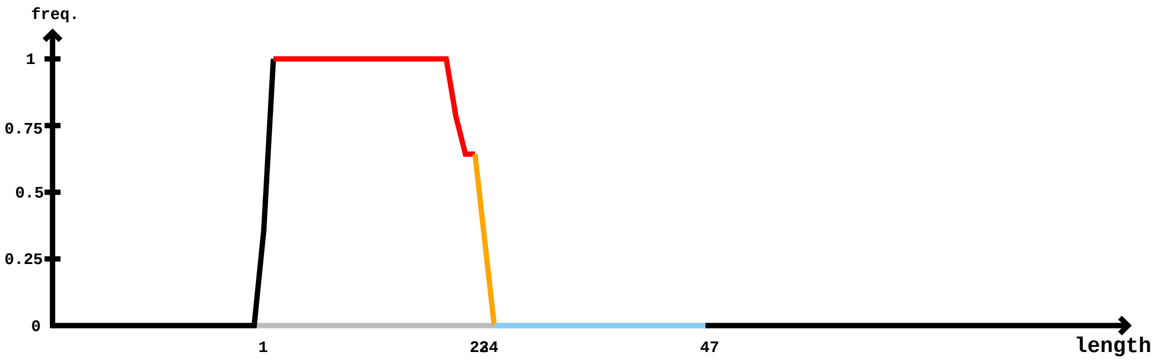


```
                 Mature         Star
5'- cuaucuauuggagcuggccccaaaaguuuguucggguuuuucuauaagauccugaaugaacuuuuggggccaacuccaguauaaccaacauaaaacauuagugagauagc   -3'   exp
    (((((((((((((.((((((((((((((((((((((((((.....)))).)))))))))))))))))))))).))))))).......................)))))).    reads     mm      sample
    ....................Aaaaaguuuguucggguuuu......................................................................    1         1       seq
    ....................caaaaguuuguucggguuuu......................................................................    2         0       seq
    ....................Aaaaaguuuguucggguuuuu.....................................................................    2         1       seq
    .....................aaaaguuuguucggguuuuucu...................................................................    2         0       seq
    .....................aaaaguuCguucggguuuuucu...................................................................    6         1       seq
    .....................aaaaguuuguucAgguuuuucu...................................................................    1         1       seq
```
